# Distribution Characteristics of Nitrogen-Cycling Microorganisms in Deep-Sea Surface Sediments of Western South China Sea

**DOI:** 10.3390/microorganisms12091901

**Published:** 2024-09-16

**Authors:** Xingjia Yin, Hui Chen, Kaixi Jiang, Boda Zhang, Ruohong Li, Xinzhe Zhu, Lianpeng Sun, Zhi Lin Ng, Ming Su

**Affiliations:** 1 School of Marine Sciences, Sun Yat-Sen University & Southern Marine Science and Engineering Guangdong Laboratory (Zhuhai), Zhuhai 519082, China; yinxj23@mail.sysu.edu.cn (X.Y.); chenhui27@mail.sysu.edu.cn (H.C.); jiangkaixi@bnu.edu.cn (K.J.); zhang_boda@outlook.com (B.Z.); ngzlin@mail.sysu.edu.cn (Z.L.N.); 2 Guangdong Provincial Key Laboratory of Marine Resources and Coastal Engineering, Zhuhai 519082, China; 3 College of Life Science and Technology, Hubei Engineering University, Xiaogan 432000, China; 4 Office of Laboratory Safety and Equipment Management, Beijing Normal University, Zhuhai 519087, China; 5 Center for Environmental Monitoring of Geology, Shenzhen 518034, China; 6 School of Environmental Science and Engineering, Sun Yat-sen University, Guangzhou 510006, China; lirh53@mail.sysu.edu.cn (R.L.); zhuxzh8@mail.sysu.edu.cn (X.Z.); eesslp@mail.sysu.edu.cn (L.S.)

**Keywords:** deep sea, environmental parameters, microbial community, nitrogen cycle, sediment

## Abstract

Nitrogen-cycling processes in the deep sea remain understudied. This study investigates the distribution of nitrogen-cycling microbial communities in the deep-sea surface sediments of the western South China Sea, using metagenomic sequencing and real-time fluorescent quantitative PCR techniques to analyze their composition and abundance, and the effects of 11 environmental parameters, including NH_4_^+^-N, NO_3_^−^-N, NO_2_^−^-N, PO_4_^3−^-P, total nitrogen (TN), total organic carbon (TOC), C/N ratio, pH, electrical conductivity (EC), SO_4_^2−^, and Cl^−^. The phylum- and species-level microbial community compositions show that five sites can be grouped as a major cluster, with sites S1 and S9 forming a sub-cluster, and sites S13, S19, and S26 forming the other; whereas sites S3 and S5 constitute a separate cluster. This is also evident for nitrogen-cycling functional genes, where their abundance is influenced by distinct environmental conditions, including water depths (shallower at sites S1 and S9 against deeper at sites S13, S19, and S26) and unique geological features (sites S3 and S5), whereas the vertical distribution of nitrogen-cycling gene abundance generally shows a decreasing trend against sediment depth. Redundancy analysis (RDA) exploring the correlation between the 11 environmental parameters and microbial communities revealed that the NO_2_^−^-N, C/N ratio, and TN significantly affect microbial community composition (*p* < 0.05). This study assesses the survival strategies of microorganisms within deep-sea surface sediments and their role in the marine nitrogen cycle.

## 1. Introduction

Seabed sediments are the largest microbial habitat on Earth, and extreme conditions such as high pressure, low temperature, and lack of oxygen in the deep-sea environment provide unique habitats for the survival of marine organisms. Nitrogen, as one of the biogenic elements in the marine environment, forms the material foundation for the survival of marine microorganisms [1,2]. In most oceanic regions, the availability of nitrogen is a crucial factor limiting primary productivity and the output of organic matter [3]. The evolution of geochemical and microbial processes over 3 billion years has kept the marine nitrogen cycle basically in equilibrium [4]. However, the rapid development of modern industry and agriculture has brought nitrogen-rich water bodies into the ocean worldwide, resulting in marine eutrophication, formation of substantial marine anoxic zones and other environmental problems [3]. Such anthropogenic ocean nitrogen imbalances can have significant impacts on marine ecosystem health, biodiversity, and climate change [5]. In recent decades, the discovery of new nitrogen-cycling processes has dramatically enriched the study of the marine nitrogen cycle. Still, it has also challenged the traditional view that the marine nitrogen reservoir is in a steady state [6].

The marine nitrogen cycle is a multi-biogeochemical process mediated by microorganisms, mainly including marine nitrogen fixation, transformations of nitrogen forms (nitrification, assimilatory and dissimilatory nitrate reduction to ammonia), and nitrogen loss (denitrification and anammox) [6,7]. Affected by the difference in seawater properties and regional environments in different periods, sediments show different vertical geochemical properties [8,9], and this vertical gradient of sediments becomes an ideal object for the study of microbial niche differentiation for the nitrogen cycle [10,11,12]. Current research shows that nitrogen-cycling microorganisms in marine sediments also exhibit vertical distribution differentiation. In the West Philippine Basin (water depth 4941.9 m), the abundance of denitrifying genes (*nirS*, *nirK*, and *nosZ*) peaked at 0–5 cm of the surface layer sediments that decreased with depth [10]. In the Ogasawara Trench sediments (water depth 9860 m), the abundance of the anammox gene increased from 1.8 × 10^5^ copies mL^−1^ at the surface layer (5 cm) to 1.2 × 10^6^ copies mL^−1^ at 15 cm, then decreased to 10^4^ copies mL^−1^ at 25 cm depth [11]. Most studies indicated that the abundance of nitrogen-cycling microorganisms declines with increasing sediment depth. However, in a Mid-Atlantic Ridge 50 m sediment core, AOA *amoA*, *nirS* and *nosZ* genes exhibited increased abundance at specific sediment depths [12]. This suggests that microorganisms might have different ecological niches in the vertical space. Strengthening the research on microbial communities in vertical surface sediments can provide deeper insights into the nitrogen-cycling processes in marine sediments.

Previously, it was thought that the factors limiting the nitrogen-cycling biosphere in deep-sea sediments were due to the lack of nutrients and energy substrates. With the increasing exploration of marine environments, other aspects such as the dissolved oxygen, temperature, pressure, pH, salinity, and sediment porosity were found to be limiting factors [13,14,15]. The seafloor is rich in fluid activity, such as pockmarks, mud volcanoes, and hydrocarbon seeps, which can bring nitrogen-cycling microorganisms from deeper to shallower layers. This process results in the formation of new community compositions and competition mechanisms in the shallower layers [16,17,18]. Under the stress of extreme environments in the deep ocean, nitrogen-cycling microorganisms have developed various adaptive mechanisms.

It is recognized that the microbial nitrogen cycle in marine sediments at different sediment depths plays an important role in the global material cycle and shows great vertical differentiation [19,20,21]. This study focuses on the microbial nitrogen cycle system in the columnar sediments of the western South China Sea, which provides new insights into the cycle process and ecological evolution of nitrogen-cycling microorganisms in deep-sea sediments through molecular biological methods through, firstly, exploring the community abundance, composition and functional distribution characteristics of nitrogen-cycling microorganisms in the deep sea at the vertical depth of surface sediments; secondly, clarifying the ecological status of nitrogen-cycling microorganisms in the biogeochemical cycle process of the deep sea; and thirdly, revealing the key environmental factors that affect the community composition. The vertical differentiation of nitrogen-cycling microorganisms is explained from the perspective of environmental science, providing important basic data and references for further exploring the global process of the marine nitrogen cycle.

## 2. Materials and Methods

### 2.1. Sampling Site Description and Sample Collection

**Figure 1:**
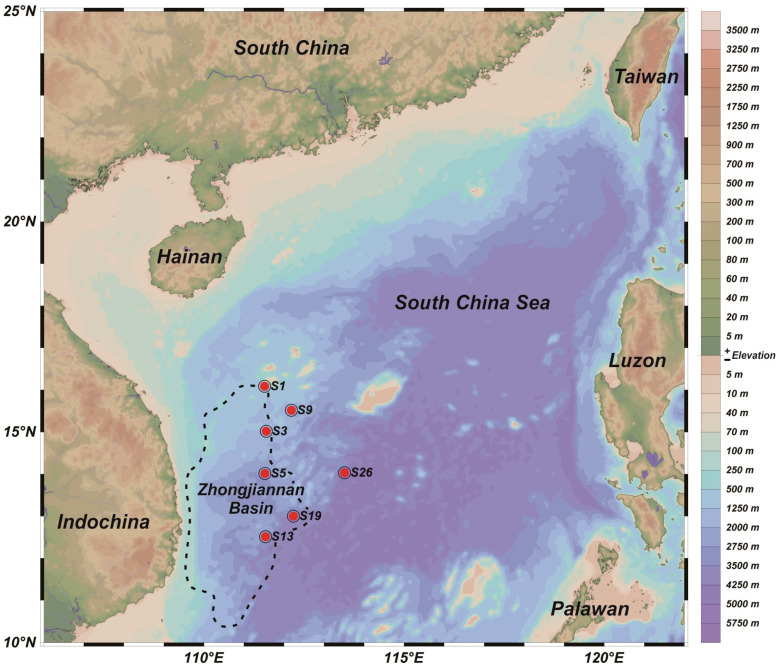
Map of the sites sampled in the western South China Sea.

From the 10th to 23th of August 2021, seven gravity cores were collected from the Zhongjiannan Basin, located in the western South China Sea, using a gravity piston sampler aboard RV Xiangyanghong 03. The distribution of the sampling sites is shown in Figure 1 and Table 1. After the sampling operation was completed, the sediment samples were stored in the sample room at −20 °C until they were transported back to the laboratory. One portion of each sample was stored at 4 °C for the environmental parameter determinations, and another portion was stored at −80 °C for DNA extraction. To study the variation of nitrogen-cycling function gene abundance in the surface sediments at different water depths, sediments from the top 30 cm were selected and sampled at every 2 cm, totaling 105 samples. To explore the composition of the microbial communities, metagenomic sequencing was performed on the mixed sediment samples from each site.

### 2.2. Environmental Parameter Analyses

The sediment samples were centrifuged at 5000 rpm for 30 min. The supernatant was then filtered through a 0.45 μm Millipore filter to obtain pore water. A total of 105 pore water samples were obtained. The concentrations of inorganic nitrogen (NH_4_^+^-N, NO_3_^−^-N, NO_2_^−^-N) and PO_4_^3−^-P in the pore water were determined by a HP 8453 UV-visible spectrophotometer (Hewlett-Packard, Palo Alto, CA, USA). The total nitrogen (TN) content and total organic carbon (TOC) content for each sediment sample were measured using a Multi 3100 TOC/TN analyzer (Jena Analytical, Jena, Germany), and the C/N ratios were then calculated. The pH and electrical conductivity (EC) of the pore water were directly measured using a FE28-Standard pH meter (Mettler Toledo, Greifensee, Switzerland) and a FE38-Standard conductivity meter (Mettler Toledo, Greifensee, Switzerland). The concentrations of SO_4_^2−^ and Cl^−^ in the pore water were determined using an ICS-3000 ion chromatograph (Dionex, Temecula, CA, USA).

### 2.3. DNA Extraction and Metagenomic Sequencing

DNA was extracted from the sediment samples using the Fast DNA Spin Kit for Soil (MP bio, Irvine, CA, USA) according to the manufacturer’s instructions. The concentrations of the extracted DNA were measured using a Nanodrop 2000 spectrophotometer (Thermo Fisher Scientific, Waltham, MA, USA), and the quality of the DNA was assessed by 1.2% agarose gel electrophoresis. Only the DNA that met the quality standards was used for the subsequent molecular experiments, where it was subjected to metagenomic sequencing and the raw reads were generated. SeqPrep (https://github.com/jstjohn/SeqPrep accessed on 22 April 2023) and Sickle (version 1.33, https://github.com/najoshi/sickle accessed on 22 April 2023) were then used to assemble the sequences. The open reading frames (ORFs) were predicted using MetaGene software (http://metagene.cb.ku-tokyo.ac.jp/ accessed on 7 June 2022), and ORFs with a length of ≥150 bp were translated into amino acid sequences based on the NCBI database. The predicted genes were clustered using CD-HIT (version 4.8.1, http://www.bioinformatics.org/cd-hit/ accessed on 7 August 2023) software (with sequence identity ≥ 95% and coverage ≥ 90%). The longest sequence from each cluster was selected as a representative to eliminate redundancy and to enhance the sequence analysis performance, thereby constructing a non-redundant gene catalog. Using the BLASTP (version 2.2.31+, http://blast.ncbi.nlm.nih.gov/Blast.cgi accessed on 15 October 2023), homologous BLAST comparisons were conducted between the non-redundant Unigenes sequences and the NCBI-NR database, setting the threshold at an e-value ≤ 0.0001 to obtain the BLAST results. The sequencing data were uploaded to the NCBI database under BioProject number PRJNA1159479.

### 2.4. Quantitative Analysis of Nitrogen-Cycling Functional Genes

The QuantStudio™ 5 real-time fluorescent quantitative PCR system (ABI, Los Angeles, CA, USA) was used for quantitative analysis of nitrogen-cycling genes. The primers used in this study are listed in Table S2, and they were mixed with the SYBR^®^ Select Master Mix (2×) kit (ABI, Los Angeles, CA, USA) to prepare a 30 μL reaction system. This amplification system included 15 μL of 2× Taqmix, 1 μL of forward primer (10 pmol μL^−1^), 1 μL of reverse primer (10 pmol μL^−1^), 1 μL of DNA template, and 12 μL of ddH_2_O. Plasmids were used as positive controls, and the PCR amplification products of the functional genes were purified and cloned. Positive recombinant plasmid DNA was extracted, with its concentration measured to calculate the copy number. Diluted recombinant plasmids were used as standard curves for detection with the qPCR instrument. Each PCR reaction included a negative control, and the standard curve and all the test samples were run in triplicate. In this study, the melting curve showed a single peak, with amplification efficiencies ranging from 91.56% to 102.45%, and the R^2^ of the standard curves ranged from 92.5% to 95.61%.

### 2.5. Statistical Analysis

The software IBM SPSS version 20.0 (SPSS Inc., Chicago, IL, USA) was used for the one-way analysis of variance (ANOVA) and Tukey’s test. Significance was evaluated by calculating the *p*-values, with *p* < 0.05 indicating a significant difference. Origin 9.0 (Origin Lab Corporation, Northampton, MA, USA) was used for plotting. A redundancy analysis (RDA) through the Monte Carlo permutation test with 499 permutations was conducted using the software CANOCO version 5 to reveal the interaction between gene abundance and environmental parameters, as well as to identify the primary factors that significantly affect the abundance of nitrogen-cycling genes, where the data were confirmed to follow a normal distribution by log-transformation before the Monte Carlo permutation test was performed [22,23]. The correlation network heatmap of nitrogen-cycling functional communities and environmental parameters was analyzed and plotted using the R version 4.1.3 software, with the correlation analysis conducted via the psych package version 2.4.6.26, Mantel test using the vegan package version 2.6-6.1, and visualization through the linkET package version 1.0 and ggplot2 package version 3.5.1.

## 3. Results

### 3.1. Sediment Environmental Properties

Table 2 shows the average values of the environmental parameters at all seven sites. The results of the difference analysis indicated that, except for the content of NO_3_^−^-N, all the other parameters were significantly different among the sites (*p* < 0.05). The NH_4_^+^-N contents in the sediments at sites S5, S13, and S26 were significantly higher than at the other sites (*p* < 0.05), with site S13 having the highest average value (292.07 μmol L^−1^) and site S3 the lowest (95.68 μmol L^−1^). The highest NO_2_^−^-N content was observed at site S5 (8.92 μmol L^−1^), while the lowest was at site S13 (3.43 μmol L^−1^). Site S13 also had the lowest NO_3_^−^-N content (3.13 μmol L^−1^), whereas the highest NO_3_^−^-N content was found at site S26 (3.13 μmol L^−1^). The highest PO_4_^3−^-P content was observed at site S9 (459.66 μmol L^−1^). The lowest contents of Cl^−^, SO_4_^2−^, TOC, and TN were found at site S5, measuring 1262.35 mmol L^−1^, 57.74 mmol L^−1^, 2.66%, and 0.67%, respectively. The highest values for SO_4_^2−^ (120.71 mmol L^−1^), TOC (10.20%), and C/N ratio (6.43) were found at site S19. The highest pH value was at site S3 (7.77) and the lowest at site S26 (7.48). However, the EC of site S26 was the highest at 32.78 mS cm^−1^. In this study, the C/N ratios were less than 10, indicating that the organic matter mainly originated from marine sources [24].

### 3.2. Microbial Community Composition in Surface Sediments of Western South China Sea

#### 3.2.1. Phylum Level

**Figure 2:**
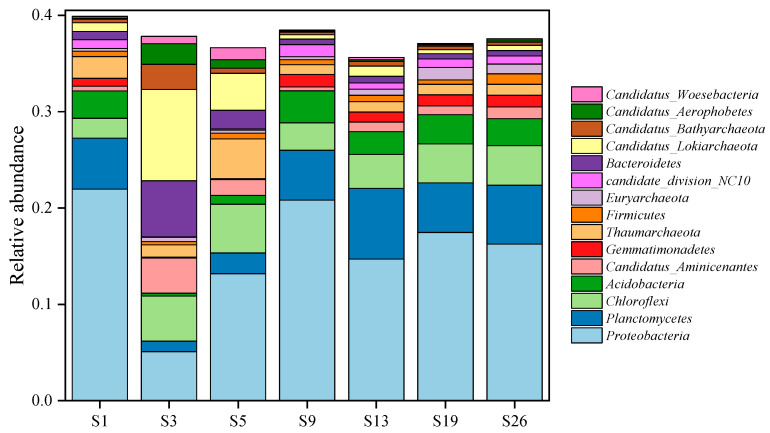
Phylum-level community composition in sediment samples from the western South China Sea (relative abundance > 1%).

The community composition at the phylum level in sediments from the seven sites is shown in Figure 2. In all of the samples, *Proteobacteria* is the dominant phylum. The community compositions of sites S1, S9, S13, S19, and S26 are similar, with *Proteobacteria*, *Planctomycetes*, *Chloroflexi*, and *Acidobacteria* being the dominant phyla. The relative abundances of *Proteobacteria* at these five sites are the highest, with relative abundances of 21.95%, 20.81%, 14.68%, 17.45%, and 16.25%, respectively. Among the other phyla, the relative abundances of *Planctomycetes* are 5.28%, 5.18%, 7.34%, 5.15%, and 6.12%; of *Chloroflexi* are 2.08%, 2.85%, 3.53%, 4.04%, and 4.10%; and of *Acidobacteria* are 2.84%, 3.33%, 2.36%, 3.04%, and 2.81%.

The community compositions at sites S3 and S5 differed noticeably from the other five sites. At site S3, the dominant phyla are *Candidatus Lokiarchaeota* (9.45%), *Bacteroidetes* (5.82%), *Proteobacteria* (5.07%), and *Chloroflexi* (4.67%). However, the dominant phyla at site S5 are *Proteobacteria* (13.17%), *Chloroflexi* (5.05%), *Thaumarchaeota* (4.13%), and *Candidatus Lokiarchaeota* (3.83%).

#### 3.2.2. Species Level

**Figure 3:**
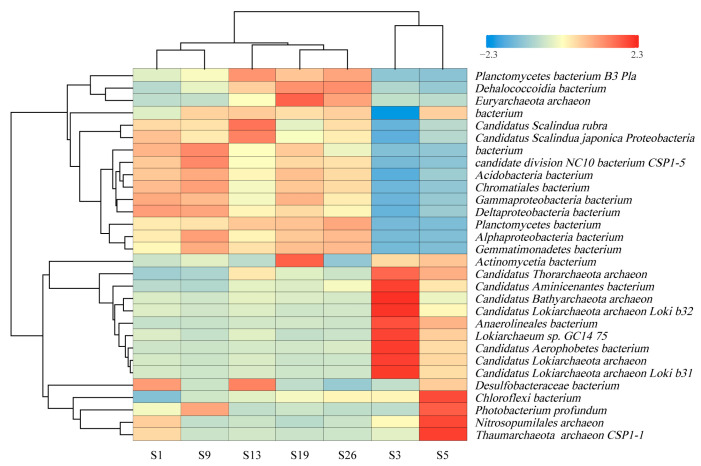
Heatmap of microbial species in sediment samples from the western South China Sea.

The top 30 key species were selected for the generation of a hierarchical cluster heatmap (Figure 3), where the colors on the heatmap represent the relative abundance of species, with blue indicating lower abundance and red indicating higher abundance. The heatmap reveals that the species composition at sites S1, S9, S13, S19, and S26 is generally similar and belongs to a major cluster, with most of their dominant species being bacteria. When subdivided further, sites S1 and S9 form a sub-cluster, while sites S13, S19, and S26 form another sub-cluster. In contrast, the microbial communities at sites S3 and S5 are markedly different from these five sampling sites. Site S3 is primarily dominated by archaea, with the dominant species being the *Lokiarchaeota archaeon*. At site S5, both bacteria, such as *Chloroflexi bacterium* and *Photobacterium profundus*, and archaea, such as *Nitrosopumilales* and *Thaumarchaeota CSP1-1*, are dominant.

### 3.3. Nitrogen-Cycling Genes Abundance in Deep-Sea Surface Sediments of Western South China Sea

#### 3.3.1. Nitrogen Fixation

**Figure 4:**
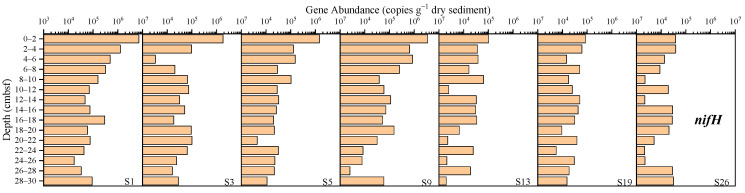
The abundance of the *nifH* gene in the sediments from the western South China Sea.

The abundance of the *nifH* gene ranged from 1.98 × 10^3^ to 7.17 × 10^6^ copies g^−1^ dry sediment (Figure 4), and the average abundance at sites S1, S3, S5, S9, S13, S19 and S26 was, respectively, 6.81 × 10^5^, 1.69 × 10^5^, 1.40 × 10^5^, 3.91 × 10^5^, 2.76 × 10^4^, 3.31 × 10^4^ and 1.81 × 10^4^ copies g^−1^ dry sediment, with the highest abundance at site S1 and the lowest abundance at site S26. Overall, the abundance levels were relatively low, with higher levels at sites S1, S3, S5 and S9 compared to sites S13, S19 and S26.

#### 3.3.2. Nitrification

**Figure 5:**
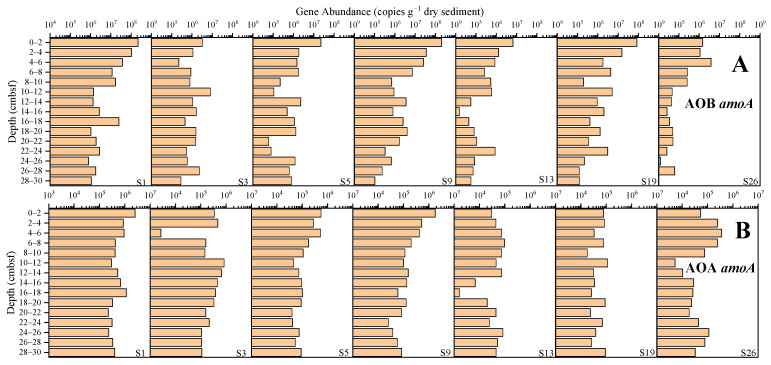
The abundance of the AOB *amoA* and AOA *amoA* genes in the sediments from the western South China Sea. ((**A**) the abundance of AOB *amoA* gene; (**B**) the abundance of AOA *amoA* gene).

The abundance of the AOB *amoA* gene ranged from 6.68 × 10^3^ to 2.18 × 10^8^ copies g^−1^ dry sediment (Figure 5A), and the average abundance at sites S1, S3, S5, S9, S13, S19 and S26 was, respectively, 2.85 × 10^7^, 1.61 × 10^6^, 2.41 × 10^6^, 1.90 × 10^7^, 7.71 × 10^5^, 7.89 × 10^6^ and 4.88 × 10^5^ copies g^−1^ dry sediment, with site S1 having the highest abundance and site S26 the lowest abundance. The abundance of the AOA *amoA* gene ranged from 1.63 × 10^3^ to 2.56 × 10^6^ copies g^−1^ dry sediment (Figure 5B), and the average abundances at sites S1, S3, S5, S9, S13, S19 and S26 was, respectively, 6.46 × 10^5^, 2.96 × 10^5^, 1.58 × 10^5^, 2.61 × 10^5^, 4.78 × 10^4^, 5.59 × 10^4^, and 9.09 × 10^4^ copies g^−1^ dry sediment, with site S1 having the highest abundance and site S13 the lowest abundance. Overall, the abundance of the AOB *amoA* gene was higher than that of the AOA *amoA* gene.

#### 3.3.3. Dissimilatory Nitrate Reduction to Ammonium (DNRA)

**Figure 6:**
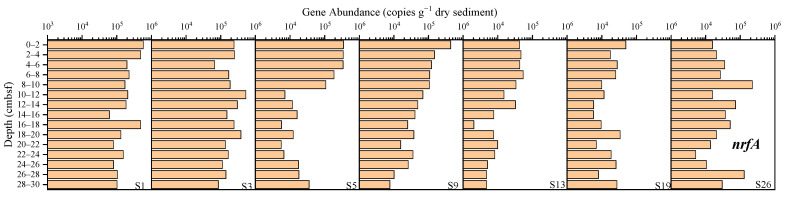
The abundance of the *nrfA* gene in the sediments from the western South China Sea.

The abundance of the *nrfA* gene ranged from 2.04 × 10^3^ to 5.79 × 10^5^ copies g^−1^ dry sediment (Figure 6). The average abundance at sites S1, S3, S5, S9, S13, S19 and S26 was, respectively, 2.17 × 10^5^, 2.11 × 10^5^, 9.74 × 10^4^, 8.28 × 10^4^, 2.14 × 10^4^, 1.89 × 10^4^, and 4.73 × 10^4^ copies g^−1^ dry sediment, with site S1 having the highest abundance and site S19 the lowest abundance. An obvious decreasing trend was observed at site S9 and S13.

#### 3.3.4. Denitrification

**Figure 7:**
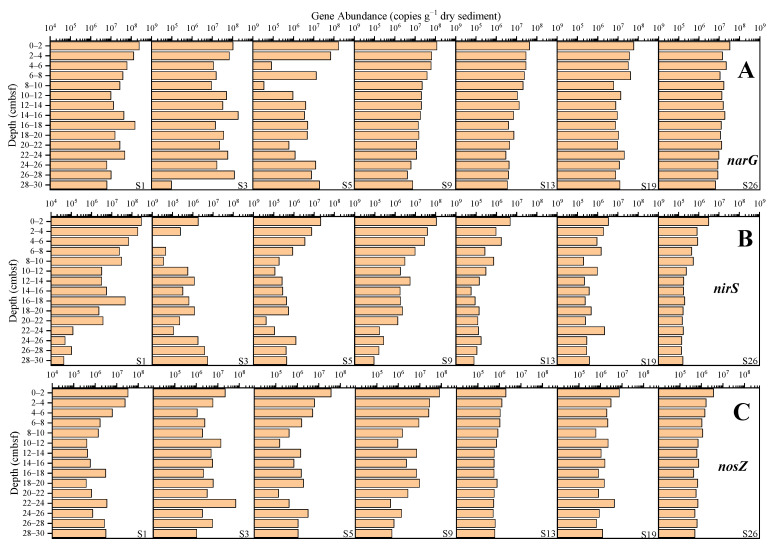
The abundance of the *narG*, *nirS*, and *nosZ* genes in the sediments from the western South China Sea. ((**A**) the abundance of *narG* gene; (**B**) the abundance of *nirS* gene; (**C**) the abundance of *nosZ* gene).

The abundance of the *narG* gene ranged from 3.51 × 10^4^ to 2.45 × 10^8^ copies g^−1^ dry sediment (Figure 7A), and the average abundance at sites S1, S3, S5, S9, S13, S19 and S26 was, respectively, 5.85 × 10^7^, 4.97 × 10^7^, 2.04 × 10^7^, 2.90 × 10^7^, 1.40 × 10^7^, 1.91 × 10^7^, and 1.44 × 10^7^ copies g^−1^ dry sediment, with the highest abundance at site S1 and the lowest at site S13. The abundance of the *nirS* gene ranged from 8.02 × 10^3^ to 2.88 × 10^8^ copies g^−1^ dry sediment (Figure 7B), and the average abundance at sites S1, S3, S5, S9, S13, S19 and S26 was, respectively, 4.43 × 10^7^, 1.18 × 10^6^, 2.45 × 10^6^, 1.35 × 10^7^, 6.52 × 10^5^, 9.08 × 10^5^, and 4.97 × 10^5^ copies g^−1^ dry sediment, with the highest abundance at site S1 and the lowest abundance at site S26. The abundance of the *nosZ* gene ranged from 1.33 × 10^5^ to 8.10 × 10^7^ copies g^−1^ dry sediment (Figure 7C), and the average abundance at sites S1, S3, S5, S9, S13, S19 and S26 was, respectively, 5.50 × 10^6^, 9.75 × 10^6^, 4.12 × 10^6^, 1.19 × 10^7^, 8.23 × 10^5^, 2.03 × 10^6^ and 9.95 × 10^6^ copies g^−1^ dry sediment, with the highest abundance at site S9 and the lowest abundance at site S13. Among the three denitrifying functional genes, *narG* showed the highest abundance levels, followed by *nirS*, with *nosZ* being the least abundant. A noticeable decreasing trend was observed for sites S9, S13 and S26.

#### 3.3.5. Anaerobic Ammonium-Oxidizing Bacteria (Anammox)

**Figure 8:**
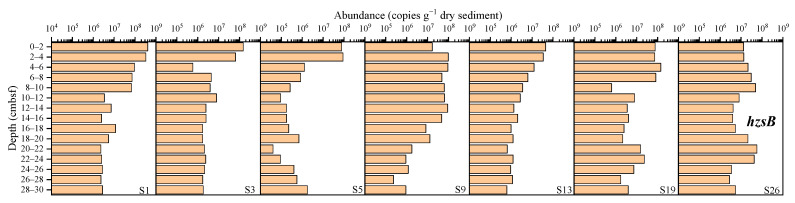
The abundance of the *hzsB* gene in the sediments from the western South China Sea.

The abundance of the *hzsB* gene ranged from 4.00 × 10^4^ to 4.05 × 10^8^ copies g^−1^ dry sediment (Figure 8), and the average abundances at sites S1, S3, S5, S9, S13, S19 and S26 was, respectively, 6.75 × 10^7^, 1.67 × 10^7^, 1.15 × 10^7^, 3.64 × 10^7^, 7.57 × 10^6^, 2.97 × 10^7^, and 1.88 × 10^7^ copies g^−1^ dry sediment, with the highest abundance at site S1 and the lowest abundance at site S13. Overall, the abundance of the *hzsB* functional gene showed a higher trend in the surface layer than in the deeper layer.

The results were analyzed comprehensively. The gene abundance at sites S13, S19, and S26 was generally low. Specifically, site S13 showed the lowest abundance of the AOA *amoA*, *narG*, *nosZ*, and *hzsB* genes, site S19 had the lowest abundance of the *nrfA* gene, and site S26 exhibited the lowest abundance of the *nifH*, AOB *amoA*, and *nirS* genes. In contrast, sites S1 and S9 had higher gene abundances. Site S1 showed the highest abundance of the *nifH*, AOA *amoA*, AOB *amoA*, *nrfA*, *narG*, *nirS*, and *hzsB* genes, while site S9 exhibited the highest abundance of the *nosZ* gene. The results showed that the abundance of nitrogen-cycling genes in the sediments from shallower water depths (sites S1 and S9) was higher than in those from deeper water depths (sites S13, S19, and S26).

### 3.4. Effects of Environmental Parameters on the Abundance of Nitrogen-Cycling Genes in the Surface Sediments of the Western South China Sea

**Figure 9:**
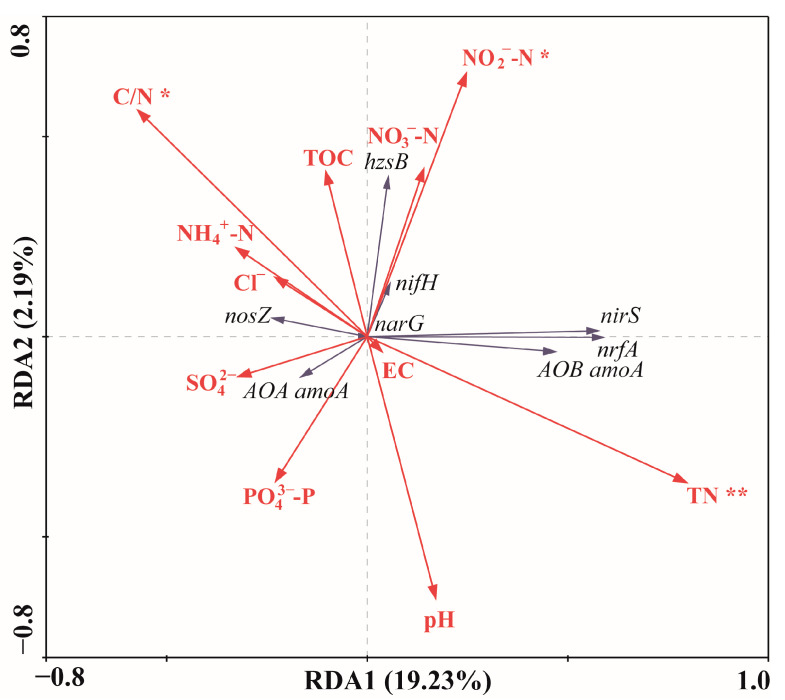
RDA of nitrogen-cycling genes and environmental parameters in the sediments from the western South China Sea. (The significance was labeled as *, *p* < 0.05; **, *p* < 0.01).

A redundancy analysis (RDA) was used to examine the relationship between the abundance of nitrogen-cycling functional genes and the environmental parameters (Figure 9). The longer arrows of environmental parameters in the RDA plot indicate a more significant influence on the species abundance variations across the dataset. The RDA results suggest that the C/N ratio (*p* = 0.043; *F* = 3.53; 499 Monte Carlo permutations), NO_2_^−^-N (*p* = 0.024; *F* = 5.30; 499 Monte Carlo permutations), and TN (*p* = 0.004; *F* = 9.95; 499 Monte Carlo permutations) were the major environmental parameters affecting the nitrogen-cycling microbial community, while the remaining environmental parameters were not significant (*p* > 0.05). From the perspective of the relationships between genes, there was a strong positive correlation among the AOB *amoA*, *nirS*, and *nrfA* (*p* < 0.01), and a significant positive correlation between *hzsB* and *nifH* (*p* < 0.01). In terms of the relationship between the environmental parameters and genes, EC was significantly negatively correlated with *nosZ* (*p* < 0.01). TN showed a significant positive correlation with *nrfA*, AOB *amoA*, and *nirS* (*p* < 0.01), while C/N was significantly negatively correlated with these three genes (*p* < 0.01).

**Figure 10:**
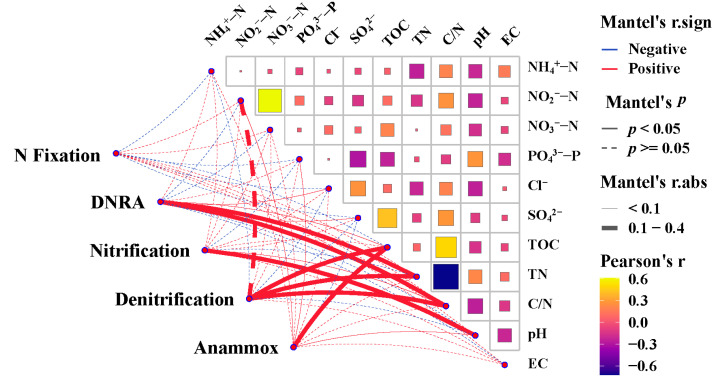
Correlation network heatmap of the gene abundance of nitrogen fixation, DNRA, nitrification, denitrification and anammox with environmental parameters.

The Mantel test assessed the impact of individual environmental parameters on the entire nitrogen-cycling microbial community (Table S3). The correlation heatmap reflects the association between nitrogen-cycling processes and environmental parameters (Figure 10). The Pearson correlation results of the environmental parameters are shown in Table S3, and the results of the Mantel test are presented in Table S4. The squares represent the correlation between environmental parameters, with yellow indicating a high correlation and blue indicating a low correlation. The lines represent the association between environmental parameters and nitrogen-cycling processes, with red lines indicating positive correlation and blue lines indicating negative correlation; solid lines represent significant correlations (*p* < 0.05), while dashed lines represent non-significant correlations (*p* > 0.05); thick lines indicate strong correlations, and thin lines indicate weak correlations. The TOC content showed a significant positive correlation with the abundance of anammox and denitrification (*p* < 0.05). TN and the C/N ratio showed significant positive correlation with DNRA and denitrification (*p* < 0.05), likewise for the pH with nitrification (*p* < 0.05).

## 4. Discussion

Metagenome sequencing was performed at the phylum and species levels to characterize the major compositions of the microbial community in deep-sea surface sediments in order to explain their variability within distinct deep-sea environments. At the phylum level, *Proteobacteria*, *Planctomycetes*, and *Chloroflexi* were of higher relative abundance in each of the seven sites in this study (Figure 2), which is consistent with the results of many marine environments, including deep-sea sediments of hypersaline anoxic basins [25], Scotian Basin cold seeps [26] and equatorial Pacific [27]. According to previous studies, most nitrifying and denitrifying bacteria (e.g., *Thauera, Denitratisoma,* and *Geobacter*) belong to *Proteobacteria* [28]. *Candidatus Lokiarchaeota*, the absolute dominant phylum at site S3, can be widely found in various marine seafloor environments [26,29,30,31]. Site S5 also has a high relative abundance of *Candidatus Lokiarchaeota*, but in addition, *Thaumarchaeota*, being its other dominant phylum, can oxidize ammonia (NH_3_) by converting it to nitrite (NO_2_^−^), being an essential part of the nitrogen cycle [32]. On the other hand, the microbial species-level cluster analysis delineated the seven sites into two separate clusters: the first, with two sub-clusters, respectively, sites S1 and S9, and sites S13, S19 and S26; the second, consisting of sites S3 and S5 (Figure 3). The species compositions of the two sub-clusters within the first major cluster are relatively similar. The second cluster, however, exhibits markedly divergent species compositions in comparison.

This classification is likely attributed to distinct geological influences for the respective clusters. Yin et al. [33] categorized the Zhongjiannan Basin into three sections based on significant differences in the structural characteristics due to tectonic dynamics: northern, central, and southern sections. The northern section comprises the Xisha Uplift, the central section features stable geological structures, while the southern section is characterized by magmatic activity. This tripartite classification is aligned with the clustering results. Sites S1 and S9 are located in the northern Zhongjiannan Basin, sites S3 and S5 in the middle Zhongjiannan Basin, and sites S13, S19, and S26 in the southern Zhongjiannan Basin. By contrast, the significant difference in species composition between S3 and S5 is likely linked to its proximity to distinct geological environments, such as pockmarks or mud volcanoes. Both sampling points, sites S3 and S5, are, respectively, situated in areas containing numerous pockmarks or mud volcanoes [34,35,36]. Pockmarks and mud volcanoes potentially release substantial amounts of organic matter, serving as a rich nutrient source for microorganisms [16,37,38,39]. They may also emit high concentrations of sulfides and heavy metals, which are toxic to certain microbial communities [40,41]. Consequently, the microbial composition in pockmarks and mud volcanoes significantly differs from that in other deep-sea environments, owing to their unique geological and chemical characteristics [42].

This spatial variation is also evident in the abundance of nitrogen cycle functional genes. The results of this study indicate that the abundance of nitrogen-cycling genes in sediments from shallower water depths (sites S1 and S9) was higher than in those from deeper water depths (sites S13, S19, and S26). Previous marine microbial studies have also demonstrated that the water depth is a significant factor influencing microbial abundance within sediments [43]. Increasing water depth is typically associated with a reduction in light intensity, lower oxygen content, decreasing temperature, higher seawater pressure, and diminution of organic matter, which are vital elements for microbial survival [44]. These factors collectively contribute to a reduced abundance of nitrogen-cycling genes in marine sediments with increasing water depth. However, it is worth noting that while site S5 (at a water depth of 2855 m) was deeper than site S13 (at a water depth of 2522 m), its overall gene abundance was higher. This discrepancy is likely due to the location of site S5, being in a unique area characterized by pockmarks and mud volcanoes. The substances released from these features, including organic matter and various chemicals, likely enhanced the activity of nitrogen-cycling microorganisms, and hence the abundance of their functional genes at site S5.

The abundance of nitrogen-cycling genes tends to decrease with the increasing vertical depth of the surface sediments, being more significant for nitrification, denitrification, and anammox functional genes. This trend, which is observed in various marine environments [10,11,45], is due to, firstly, the reduction in TOC (Table S1), which acts as an energy source for microbial heterotrophic processes, inhibiting denitrification; and secondly, potential higher ammonia oxidation activity at the surface, as an important control for the nitrification process [10]. Higher anammox rates can also be inferred for shallower surface sediments, as it positively correlates with anammox gene abundance [10], whereas other environmental parameters that exhibit positive correlation with individual nitrogen-cycling processes, including TN, C/N, and pH, do not show any statistically significant trend with the sediment depth (Figure 10). In this study, the most notable decrease was observed at site S9, with sites S13, S19, and S26 also showing some decreasing trends in specific genes. However, sites S1, S3, and S5 exhibited significant fluctuations in gene abundance. For example, an increase was observed at the 12–18 cm segment at site S1, affecting almost every gene. At site S3, a sharp decrease was noted at the 4–6 cm segment, whereas at the 10–12 cm and 20–22 cm segments of site S5, almost all the genes showed dramatic changes. This variability likely stems from the faster ocean current flow and sediment accumulation at sites S1, S3, and S5, leading to significant differences in sediment properties. In contrast, the lower flow rates and slower sediment accumulation at sites S9, S13, S19, and S26 result in more stable sediment properties and less dramatic changes in microbial communities.

In this study, factors significantly affecting the microbial community included the C/N ratio *(p* < 0.05), NO_2_^−^-N (*p* < 0.05), and TN (*p* < 0.01). Carbon and nitrogen are fundamental nutrients for microbial growth, and their relative ratio directly influence microbial metabolic activities and growth. Wu et al. [46] found that the C/N ratio significantly affects the abundance of *amoA*, *nirS*, *nirK*, and *nosZ* genes in lake sediments. Similarly, Fu et al. [45] reported that AOA, AOB, SRB, and denitrifying bacteria are positively correlated with TOC, TN, and the C/N ratio in the Yap Trench (*p* < 0.01). The influence of NO_2_^−^-N on nitrogen-cycling genes has been confirmed in various environments, especially on *hzsB* and *nirS* genes, as NO_2_^−^-N serves as a substrate for reactions regulated by these genes. Consequently, the abundance of these genes is often positively correlated with NO_2_^−^-N. However, competition may arise when the substrate is insufficient, resulting in varying relationships in different environments. In this study, NO_2_^−^-N is significantly correlated with *hzsB* abundance (*p* < 0.01) but not with *nirS* (*p* > 0.05), indicating that NO_2_^−^-N favors the growth of anammox bacteria over denitrifying bacteria in the competition between *hzsB* and *nirS* genes.

## 5. Conclusions

This research reveals the clustering of microbial community composition and the relationship between the abundance of nitrogen-cycling functional genes and environmental parameters in the deep-sea surface sediments of the western South China Sea. The analysis indicates that NO_2_^−^-N, C/N ratio, and TN are essential factors influencing nitrogen-cycling microbial communities. Overall, the nitrogen-cycling genes showed a decreasing trend, with higher abundance in shallower water depths compared to deeper water depths. Also, the abundance of nitrogen-cycling genes at sites S3 and S5 was markedly different from that at other sampling points, suggesting the possible influences of unique geological features at these two locations. This is supported by the phylum-level community composition and species-level clustering analysis of the composition of the microbial communities. Additionally, the vertical distribution of nitrogen cycle functional genes generally shows a decreasing trend with the sediment depth, where their abundance is generally controlled by their respective environmental parameters. Therefore, this study investigates the distribution characteristics of various nitrogen-cycling microorganisms, providing insights into the survival strategies of these microbes in deep-sea basins, which is crucial for understanding the balance of marine nitrogen cycles.

## Figures and Tables

**Table 1:** Sampling locations and water depths of sediments from the western South China Sea.

Site	Latitude	Longitude	Water Depth (m)
S1	16.09° N	111.52° E	1179
S3	15.02° N	111.56° E	1395
S5	14.02° N	111.52° E	2855
S9	15.51° N	112.19° E	1889
S13	12.51° N	111.54° E	2522
S19	13.00° N	112.24° E	3563
S26	14.03° N	113.51° E	4150

**Table 2:** Environmental parameters in deep-sea surface sediments from the western South China Sea.

Site	NH_4_^+^-N	NO_2_^−^-N	NO_3_^−^-N	PO_4_^3−^-P	Cl^−^	SO_4_^2−^	TOC	TN	C/N	pH	EC
	μmol L^−1^	μmol L^−1^	μmol L^−1^	μmol L^−1^	mmol L^−1^	mmol L^−1^	%	%			mS cm^−1^
S1 (n = 15)	142.32 ± 49.67 ^ab^	4.80 ± 1.98 ^ab^	5.50 ± 6.44 ^a^	427.45 ± 192.48 ^a^	1545.34 ± 281.11 ^bc^	70.30 ± 13.67 ^cd^	7.49 ± 0.26 ^bc^	4.51 ± 0.07 ^b^	1.67 ± 0.53 ^c^	7.69 ± 0.09 ^b^	31.38 ± 1.32 ^ab^
S3 (n = 15)	95.68 ± 48.14 ^b^	4.21 ± 2.59 ^ab^	5.53 ± 2.40 ^a^	258.31 ± 130.21 ^b^	1390.74 ± 213.52 ^c^	73.81 ± 24.18 ^cd^	5.65 ± 0.12 ^d^	6.12 ± 0.18 ^a^	0.96 ± 0.18 ^c^	7.77 ± 0.10 ^a^	30.77 ± 1.41 ^b^
S5 (n = 15)	264.85 ± 145.06 ^a^	8.92 ± 3.08 ^a^	3.37 ± 3.09 ^a^	374.52 ± 214.95 ^a^	1262.35 ± 425.48 ^c^	57.74 ± 25.29 ^d^	2.66 ± 0.14 ^e^	0.67 ± 0.02 ^d^	4.04 ± 1.64 ^b^	7.70 ± 0.07 ^b^	31.58 ± 1.43 ^ab^
S9 (n = 15)	108.27 ± 29.61 ^b^	7.26 ± 13.87 ^ab^	9.60 ± 11.95 ^a^	459.66 ± 114.04 ^a^	1870.85 ± 392.73 ^ab^	75.53 ± 21.51 ^c^	6.88 ± 0.25 ^cd^	1.10 ± 0.01 ^cd^	6.28 ± 2.30 ^a^	7.70 ± 0.13 ^ab^	27.33 ± 3.69 ^c^
S1 (n = 15)	292.07 ± 254.87 ^a^	3.43 ± 1.85 ^b^	3.13 ± 1.72 ^a^	400.53 ± 90.73 ^a^	1817.42 ± 461.37 ^ab^	73.60 ± 17.23 ^cd^	8.62 ± 0.09 ^a^	1.39 ± 0.02 ^c^	6.32 ± 0.93 ^a^	7.70 ± 0.09 ^ab^	30.94 ± 1.45 ^b^
S19 (n = 15)	227.79 ± 144.58 ^ab^	5.28 ± 2.41 ^ab^	4.29 ± 2.15 ^a^	164.42 ± 47.57 ^bc^	1583.26 ± 1094.12 ^bc^	120.71 ± 24.13 ^a^	10.20 ± 0.15 ^a^	1.61 ± 0.02 ^c^	6.43 ± 1.09 ^a^	7.70 ± 0.07 ^ab^	30.30 ± 1.45 ^b^
S26 (n = 15)	267.21 ± 364.55 ^a^	6.90 ± 5.78 ^ab^	9.99 ± 24.99 ^a^	150.70 ± 60.02 ^c^	2181.61 ± 269.16 ^a^	93.74 ± 24.83 ^b^	6.51 ± 0.14 ^cd^	1.19 ± 0.02 ^cd^	5.52 ± 1.19 ^a^	7.48 ± 0.06 ^c^	32.78 ± 1.02 ^a^

Different letters after values in the same column indicate significant differences (*p* < 0.05).

## Data Availability

The original contributions presented in this study are included in the article/Supplementary Materials; further inquiries can be directed to the corresponding author.

## Supplementary Materials

The following supporting information can be downloaded at https://www.mdpi.com/article/10.3390/microorganisms12091901/s1, Table S1: Environmental characteristics of sediment samples from the western South China Sea; Table S2: Primer pairs used in this study; Table S3: The Pearson correlation results of environmental parameters in sediment samples from the western South China Sea; Table S4: Mantel test results between nitrogen-cycling functional communities and environmental parameters in sediment samples from the western South China Sea. Refs. [47,48,49,50,51,52] can be found in Supplementary Materials.
