# Supplementary material for: Distribution Characteristics of Nitrogen-Cycling Microorganisms in Deep-Sea Surface Sediments of Western South China Sea
Source: Microorganisms. 2024 Sep 16;12(9):1901. doi: 10.3390/microorganisms12091901 (PMC11434414; doi:10.3390/microorganisms12091901)
Supplement: Supplementary file 1 [file microorganisms-12-01901-s001.zip › microorganisms-3190789-supplementary.pdf]

Supplemental Data

Table S1. Environmental characteristics of sediment samples from the western South China Sea.

Sites	Depth (cmbsf)	TN (%)	TOC (%)	C/N ratio	NH_4^+-N (μmol L^{-1})	NO_2^--N (μmol L^{-1})	NO_3^--N (μmol L^{-1})	PO_4^{3-}-P (μmol L^{-1})	Cl^- (μmol L^{-1})	SO_4^{2-} (μmol L^{-1})	pH	EC (mS cm^{-1})
S1	0–2	0.45	1.25	2.79	165.25	6.08	20.40	219.74	1806.56	73.09	7.53	33.34
	2–4	0.43	0.70	1.64	133.92	3.28	5.50	237.99	1430.86	62.80	7.63	31.91
	4–6	0.42	0.67	1.59	180.77	5.24	5.85	223.72	1448.91	56.40	7.66	31.87
	6–8	0.46	0.60	1.30	83.94	7.57	19.00	404.37	1684.80	90.84	7.63	30.84
	8–10	0.48	1.29	2.66	260.31	8.86	11.05	299.70	2009.80	94.70	7.55	30.18
	10–12	0.56	0.63	1.12	90.26	3.34	3.33	581.24	1624.85	79.71	7.64	32.22
	12–14	0.54	1.18	2.17	112.61	3.66	0.83	179.90	1171.48	65.50	7.66	31.20
	14–16	0.49	0.76	1.54	234.60	7.70	6.25	476.28	1587.67	75.84	7.72	28.89
	16–18	0.47	0.66	1.41	106.16	3.51	1.02	231.20	1618.64	84.74	7.68	33.47
	18–20	0.47	0.69	1.47	116.51	5.13	0.24	348.97	1491.33	69.66	7.81	32.31
	20–22	0.34	0.68	2.00	143.72	4.66	1.44	630.00	1321.90	63.57	7.66	31.64
	22–24	0.47	0.49	1.03	132.90	4.69	1.12	527.20	1471.22	67.17	7.86	30.49
	24–26	0.48	0.60	1.24	124.75	3.44	1.14	670.68	1306.87	50.41	7.74	31.33
	26–28	0.30	0.56	1.88	121.06	2.68	2.71	711.77	2105.30	72.78	7.72	29.11
	28–30	0.41	0.51	1.23	127.94	2.18	2.56	668.99	1099.85	47.22	7.79	31.87
S3	0–2	1.01	0.73	0.72	67.52	2.64	7.79	599.23	1257.66	75.05	7.58	31.49
	2–4	0.69	0.71	1.02	47.50	4.94	3.62	97.66	1461.91	58.04	7.60	30.56
	4–6	0.82	0.74	0.90	135.99	3.77	6.40	220.23	1152.67	144.91	7.67	31.63
	6–8	0.65	0.72	1.10	56.54	5.33	6.84	153.66	1203.12	53.00	7.68	28.60
	8–10	0.68	0.63	0.93	19.86	5.28	10.34	148.24	1462.75	61.35	7.81	30.40
	10–12	0.71	0.59	0.83	160.59	3.62	7.07	79.49	1102.27	45.22	7.84	31.18
	12–14	0.77	0.53	0.68	73.77	2.56	3.23	225.02	1275.34	60.34	7.85	32.36
	14–16	0.69	0.56	0.81	99.53	3.19	3.01	192.18	1382.24	71.08	7.74	31.38
	16–18	0.35	0.46	1.33	96.87	2.20	7.96	287.78	1464.31	70.98	7.76	30.87
	18–20	0.48	0.54	1.13	46.06	7.63	2.25	330.00	1570.40	88.49	7.88	30.16
	20–22	0.46	0.47	1.03	95.44	11.70	7.75	369.63	1918.10	91.44	7.74	30.83
	22–24	0.39	0.42	1.08	105.84	2.89	5.55	284.32	1563.15	93.20	7.86	27.69

	24–26	0.47	0.51	1.09	96.77	1.35	3.96	281.98	1174.71	60.97	7.85	29.73
	26–28	0.47	0.43	0.91	126.97	2.97	4.26	372.55	1549.09	73.71	7.79	31.18
	28–30	0.56	0.45	0.80	206.01	3.09	2.94	232.72	1323.34	59.39	7.89	33.52
S5	0–2	0.04	0.20	5.50	248.95	11.14	1.08	46.52	610.01	29.03	7.59	31.42
	2–4	0.08	0.35	4.44	128.13	11.17	1.91	150.50	1178.70	48.88	7.68	31.62
	4–6	0.08	0.35	4.46	77.43	7.90	3.61	69.90	1039.72	51.04	7.73	31.79
	6–8	0.05	0.22	4.46	159.45	6.43	3.48	530.82	1647.51	72.27	7.70	32.41
	8–10	0.09	0.54	5.77	241.56	2.78	4.80	218.25	1447.42	63.94	7.76	32.77
	10–12	0.10	0.31	3.12	215.93	10.69	2.06	639.26	1482.74	70.36	7.65	34.43
	12–14	0.04	0.26	7.11	377.38	8.90	0.64	544.94	1224.07	75.15	7.76	31.70
	14–16	0.07	0.08	1.20	527.00	6.70	1.26	591.81	1346.56	64.55	7.63	29.21
	16–18	0.06	0.14	2.35	244.41	14.77	1.09	630.55	1419.54	33.73	7.68	31.11
	18–20	0.06	0.08	1.37	85.59	7.96	9.77	344.97	1331.76	37.88	7.59	29.92
	20–22	0.05	0.25	4.93	544.10	10.52	0.89	312.04	743.67	39.29	7.76	29.71
	22–24	0.06	0.17	2.63	260.22	10.29	3.70	671.78	1026.04	49.68	7.83	31.39
	24–26	0.06	0.21	3.79	273.15	7.45	9.73	218.27	735.70	35.94	7.78	32.10
	26–28	0.07	0.31	4.71	422.56	12.26	0.34	243.71	1356.94	63.43	7.64	33.72
	28–30	0.11	0.52	4.73	166.95	4.78	6.24	404.55	2344.92	131.00	7.65	30.40
S9	0–2	0.12	0.49	4.11	106.69	10.36	3.18	391.03	1507.88	63.09	7.44	19.27
	2–4	0.11	1.50	13.54	78.86	56.89	51.76	551.93	1179.21	44.87	7.50	27.24
	4–6	0.12	0.82	6.78	156.03	2.18	8.17	180.44	1370.24	56.70	7.54	29.78
	6–8	0.10	0.80	7.97	159.89	3.06	5.76	488.88	1323.24	58.28	7.66	28.71
	8–10	0.11	0.45	3.91	82.24	3.87	10.05	373.41	2011.85	66.07	7.72	23.81
	10–12	0.11	0.40	3.64	85.22	3.24	8.70	495.55	2303.42	95.05	7.78	26.75
	12–14	0.11	0.61	5.55	135.38	3.30	8.49	556.47	1831.77	51.38	7.83	32.76
	14–16	0.11	0.68	5.95	76.34	2.32	5.22	479.61	1956.20	88.74	7.82	29.46
	16–18	0.11	0.67	6.06	101.23	3.54	0.98	586.27	1666.35	64.71	7.77	30.93
	18–20	0.11	0.70	6.50	139.03	2.61	7.16	493.82	2027.26	76.83	7.67	29.33
	20–22	0.11	0.67	6.04	128.61	3.67	9.09	534.17	2001.15	96.72	7.79	28.04
	22–24	0.11	0.61	5.69	108.68	3.57	7.05	564.40	2091.80	80.61	7.68	27.42
	24–26	0.11	0.68	6.21	108.09	4.40	4.28	305.04	2178.03	77.95	7.89	21.32
	26–28	0.10	0.61	6.01	65.50	2.36	4.43	529.31	2010.74	83.07	7.65	30.53
	28–30	0.10	0.63	6.30	92.26	3.54	9.75	364.58	2603.59	128.88	7.78	24.61
S13	0–2	0.15	0.91	5.98	242.13	1.44	5.49	266.96	2107.59	75.28	7.62	32.98

	2–4	0.15	0.85	5.80	132.59	1.18	4.71	208.53	2552.27	93.38	7.66	32.23
	4–6	0.14	1.08	7.61	1073.82	1.16	4.06	395.11	1761.24	49.50	7.58	29.22
	6–8	0.15	0.90	6.06	119.57	2.79	5.09	325.74	1863.13	86.30	7.78	29.82
	8–10	0.20	1.00	4.96	612.41	3.00	5.72	391.56	1819.14	69.80	7.75	31.89
	10–12	0.15	0.82	5.42	104.94	1.69	4.69	332.73	2048.48	82.10	7.74	28.58
	12–14	0.14	0.81	5.62	75.75	2.89	2.64	400.48	1822.27	67.17	7.74	33.52
	14–16	0.13	0.92	7.16	241.19	2.71	2.27	413.28	2747.36	102.40	7.57	30.42
	16–18	0.10	0.81	7.79	264.21	4.24	2.84	526.75	2003.05	99.05	7.68	30.18
	18–20	0.13	0.80	6.20	360.69	3.97	1.25	395.59	1658.09	68.45	7.88	32.67
	20–22	0.14	0.79	5.64	280.06	6.70	1.33	470.02	1895.64	75.53	7.65	30.78
	22–24	0.13	0.86	6.48	295.74	3.72	3.33	398.51	1101.90	48.74	7.77	30.07
	24–26	0.12	0.84	6.81	224.02	3.35	1.22	451.37	1433.06	77.55	7.73	31.31
	26–28	0.11	0.86	7.85	268.60	7.15	0.82	525.82	1259.84	56.39	7.61	30.74
	28–30	0.13	0.69	5.40	85.33	5.40	1.55	505.49	1188.25	52.30	7.76	29.67
S19	0–2	0.18	0.93	5.13	200.09	11.89	3.63	279.06	2376.59	128.42	7.63	32.85
	2–4	0.16	0.93	5.67	598.79	4.93	2.90	210.68	2422.80	99.66	7.58	32.98
	4–6	0.17	1.03	6.07	94.91	2.42	6.04	153.95	278.99	122.58	7.68	29.64
	6–8	0.16	1.14	6.98	217.70	5.20	9.13	135.26	359.34	141.81	7.70	29.69
	8–10	0.16	1.15	6.98	31.04	6.23	5.79	143.58	2106.02	68.99	7.72	31.07
	10–12	0.22	1.12	5.12	285.82	9.30	0.52	121.76	298.34	97.01	7.59	30.26
	12–14	0.18	1.13	6.37	371.89	4.18	4.00	106.84	316.50	166.63	7.78	28.26
	14–16	0.18	0.79	4.41	228.59	5.32	4.76	153.04	2277.74	108.04	7.75	30.75
	16–18	0.16	1.34	8.27	89.78	5.05	4.80	123.98	284.12	126.08	7.70	29.95
	18–20	0.13	1.02	7.84	279.93	4.69	7.15	119.85	2512.00	120.28	7.79	27.95
	20–22	0.14	1.04	7.21	161.81	3.96	1.93	178.90	2853.70	108.51	7.81	29.33
	22–24	0.16	1.11	6.86	159.89	3.74	3.41	168.77	2435.87	110.17	7.63	31.91
	24–26	0.12	0.88	7.51	258.39	3.12	4.56	140.42	278.54	153.47	7.78	30.49
	26–28	0.14	0.82	5.84	363.72	5.16	2.85	224.51	2344.14	120.07	7.67	29.73
	28–30	0.14	0.87	6.23	74.45	3.96	2.87	205.72	2604.19	138.97	7.75	29.64
S26	0–2	0.14	0.82	5.98	108.01	10.19	11.50	266.59	2440.28	100.60	7.55	33.96
	2–4	0.17	0.78	4.63	346.97	25.91	99.82	288.21	2348.51	99.96	7.52	32.06
	4–6	0.14	0.62	4.52	208.38	8.68	3.65	117.80	2329.34	99.46	7.53	32.29
	6–8	0.12	0.53	4.28	151.03	8.91	1.71	132.01	2274.27	96.59	7.50	32.77
	8–10	0.09	0.60	6.48	208.36	7.18	2.20	122.95	2409.83	95.82	7.56	34.67

10–12	0.10	0.50	4.96	72.85	6.66	4.63	186.86	2499.62	125.37	7.49	33.60
12–14	0.12	0.81	7.03	307.12	7.01	4.10	127.74	2168.70	76.69	7.53	32.77
14–16	0.13	0.89	6.88	180.42	4.09	5.45	117.19	2064.88	153.14	7.42	33.48
16–18	0.12	0.55	4.65	19.35	3.06	4.66	116.65	2186.49	115.96	7.39	32.17
18–20	0.10	0.78	7.64	35.22	3.41	3.68	122.75	2384.42	84.19	7.36	31.58
20–22	0.12	0.64	5.34	1542.15	4.04	2.66	96.86	1866.64	74.96	7.38	33.84
22–24	0.12	0.70	6.09	194.25	4.04	1.83	131.33	2150.21	76.07	7.50	30.99
24–26	0.12	0.39	3.23	176.65	2.72	2.04	91.90	1461.15	58.04	7.50	32.89
26–28	0.10	0.57	5.43	182.26	3.74	0.70	129.31	1934.45	60.08	7.47	31.58
28–30	0.11	0.61	5.72	275.06	3.92	1.21	212.37	2205.34	89.15	7.53	33.12

Table S2. Primer pairs used in this study.

Target gene	Primer pair	Sequences（5'-3')	Size (bp)	Reference
nirH	nifH-F	AAAGGYGGWATCGGYAARTCCACCAC	464	[47]
	nifH-R	TTGTTSGCSGCRTACATSGCCATCAT		
AOA *amoA*	amoAF	STAATGGTCTGGCTTAGACG	230	[48]
	amoAR	GCGGCCATCCATCTGTATGT		
AOB *amoA*	amoA-1F	GGGGTTTCTACTGGTGGT	494	[48]
	amoA-2R	CCCCTCKGSAAAGCCTTCTTC		
nrfA	nrfA-2F	CACGACAGCAAGACTGCCG	230	[49]
	nrfA-2R	CCGGCACTTTCGAGCCC		
narG	narGF	TCGCCSATYCCGGCSATGTC	176	[50]
	narGR	GAGTTGTACCAGTCRGCSGAYTCSG		
nirS	cd3Af	GTSAACGTSAAGGARACSGG	413	[51]
	R3cd	GASTTCGGRTGSGTCTTGA		
nosZ	nosZF	CGYTGTTCMTCGACAGCCAG	460	[43]
	nosZR	CGSACCTTSTTGCCSTYGCG		
hzsB	HSBeta396F	ARGGHTGGGGHAGYTGGAAG	388	[52]
	HSBeta742R	GTYCCHACRTCATGVGTCTG		

References

43. Yu, T.T.; Li, M.; Niu, M.Y.; Fan, X.B.; Liang, W.Y.; Wang, F.P. Difference of nitrogen-cycling microbes between shallow bay and deep-sea sediments in the South China Sea. *Appl Microbiol Biot*. **2018,** *102*, 447-459. https://doi.org/10.1007/s00253-017-8594-9.

47. Rong, L.L.; Zhao, L.F.; Zhao, L.C.; Cheng, Z.P.; Yao, Y.M.; Yuan, C.L.; Wang, L.; Sun, H.W. LDPE microplastics affect soil microbial communities and nitrogen cycling. *Sci Total Environ*. **2021,** *773*, 145640. https://doi.org/10.1016/j.scitotenv.2021.145640.

48. Chang, Y.K.; Yin, G.Y.; Hou, L.J.; Liu, M.; Zheng, Y.L.; Han, P.; Dong, H.P.; Liang, X.; Gao, D.Z.; Liu, C. Nitrogen removal processes coupled with nitrification in coastal sediments off the north East China Sea. *J Soil Sediment*. **2021,** *21*, 3289-3299. https://doi.org/10.1007/s11368-021-02964-5.

49. Shi, Y.; Zhang, X.Y.; Wang, Z.C.; Xu, Z.W.; He, C.G.; Sheng, L.X.; Liu, H.Y.; Wang, Z.Q. Shift in nitrogen transformation in peatland soil by nitrogen inputs. *Sci Total Environ*. **2021,** *764*, 142924. https://doi.org/10.1016/j.scitotenv.2020.142924.

50. Chen, C.; Yin, G.Y.; Hou, L.J.; Liu, M.; Jiang, Y.H.; Zheng, D.S.; Gao, D.Z.; Liu, C.; Zheng, Y.L.; Han, P. Effects of sulfamethoxazole on coupling of nitrogen removal with nitrification in Yangtze Estuary sediments. *Environ Pollut*. **2021,** *271*, 116382. https://doi.org/10.1016/j.envpol.2020.116382.

51. Huang, F.J.; Lin, X.B.A.; Hu, W.F.; Zeng, F.; He, L.; Yin, K.D. Nitrogen cycling processes in sediments of the Pearl River Estuary: Spatial variations, controlling factors, and environmental implications. *Catena*. **2021,** *206*, 105545. https://doi.org/10.1016/j.catena.2021.105545.

52. Wu, J.P.; Hong, Y.G.; Chang, X.Y.; Jiao, L.J.; Li, Y.B.; Liu, X.H.; Xie, H.T.; Gu, J.D. Unexpectedly high diversity of anammox bacteria detected in deep-sea surface sediments of the South China Sea. *Fems Microbiol Ecol*. **2019,** *95*, fiz013. https://doi.org/10.1093/femsec/fiz013.

Table S3. The Pearson correlation results of environmental parameters in sediment samples from the western South China Sea.

Pearson (n=105)	NH_4^+-N	NO_2^--N	NO_3^--N	PO_4^{3-}-P	Cl^-	SO_4^{2-}	TOC	TN	C/N	pH	EC
NH_4^+-N	1.000										
NO_2^--N	0.004	1.000									
NO_3^--N	−0.023	0.613**	1.000								
PO_4^{3-}-P	−0.060	0.098	0.019	1.000							
Cl^-	−0.016	−0.088	0.091	0.004	1.000						
SO_4^{2-}	−0.050	−0.142	0.048	−0.300**	0.265**	1.000					
TOC	0.044	0.101	0.202*	−0.239*	0.088	0.422**	1.000				
TN	−0.247*	−0.153	0.005	−0.030	−0.212*	−0.089	0.065	1.000			
C/N	0.195*	0.262**	0.123	−0.104	0.192*	0.277**	0.493**	−0.726**	1.000		
pH	−0.212*	−0.235*	−0.173	0.272**	−0.246*	−0.107	−0.157	0.218*	−0.254**	1.000	
EC	0.160	−0.058	−0.065	−0.181	0.020	−0.040	−0.062	0.097	−0.131	−0.212*	1.000

The significance was labeled as *, $p < 0.05$; **, $p < 0.01$.

Table S4. Mantel test results between nitrogen-cycling functional communities and environmental parameters in sediment samples from the western South China Sea.

Environmental Parameters	*nifH*		AOB *amoA* + AOA *amoA*		*nrfA*		*narG* + *nirS* + *nosZ*		*hzsB*	
	r	*p*	*r*	*p*	*r*	*p*	*r*	*p*	*r*	*p*
NH_4^+-N	−0.0158	0.505	0.0158	0.3575	0.0119	0.295	−0.0485	0.7378	0.0511	0.125
NO_2^--N	0.0995	0.114	0.0752	0.1125	−0.0020	0.449	0.1011	0.1174	0.0953	0.024
NO_3^--N	0.0324	0.291	0.0145	0.3623	−0.0122	0.556	0.0077	0.3766	0.0479	0.159
PO_4^{3-}-P	−0.0257	0.766	−0.0347	0.8815	0.0098	0.303	0.0391	0.1446	0.0742	0.005
Cl^-	−0.0793	0.965	0.0052	0.4374	0.0642	0.017	−0.0409	0.8076	0.0289	0.196
SO_4^{2-}	−0.0602	0.891	0.0142	0.3496	0.0489	0.038	−0.0396	0.7761	−0.0031	0.509
TOC	−0.0256	0.664	0.0220	0.2884	0.0601	0.026	0.1117	0.0193	0.1542	0.001
TN	0.0757	0.083	−0.0026	0.4863	0.2794	0.001	0.1659	0.0036	0.0118	0.344
C/N	0.0487	0.14	0.0883	0.0078	0.2499	0.001	0.1117	0.0059	0.0003	0.436
pH	0.0464	0.161	0.1939	0.0001	−0.0219	0.796	−0.0257	0.6996	0.0676	0.021
EC	0.0846	0.097	0.0645	0.1087	−0.0020	0.445	0.0424	0.2233	0.0049	0.423
